# Supplementary material for: Iterative improvement in the automatic modular design of robot swarms
Source: PeerJ Comput Sci. 2020 Dec 7;6:e322. doi: 10.7717/peerj-cs.322 (PMC7924708; doi:10.7717/peerj-cs.322)
Supplement: Supplemental Information 3 [file peerj-cs-06-322-s003.zip › argos3/doc/api/standalone/a00558_source.html]

ARGoS: plugins/robots/spiri/simulator/pointmass3d\_spiri\_model.cpp Source File


- Main Page
- Related Pages
- Namespaces
- Classes
- Files

- File List
- File Members

# plugins/robots/spiri/simulator/pointmass3d\_spiri\_model.cpp

Go to the documentation of this file.

```
00001 
00007 #include "pointmass3d_spiri_model.h"
00008 #include <argos3/core/utility/logging/argos_log.h>
00009 #include <argos3/core/utility/math/cylinder.h>
00010 #include <argos3/core/simulator/simulator.h>
00011 #include <argos3/core/simulator/space/space.h>
00012 #include <argos3/plugins/simulator/physics_engines/pointmass3d/pointmass3d_engine.h>
00013 
00014 namespace argos {
00015 
00016    static const Real     BODY_HEIGHT   = 0.090f;
00017    static const Real     BODY_SIDE     = 0.470f;
00018    static const Real     BODY_DIAGONAL = BODY_SIDE * ::sqrt(2);
00019    static const Real     BODY_RADIUS   = BODY_DIAGONAL / 2.0f;
00020    static const Real     BODY_MASS     = 1.00f;
00021    static const Real     BODY_INERTIA  = 0.01f;
00022    static const CVector3 POS_K_P(20.0f, 20.0f, 20.0f);
00023    static const CVector3 POS_K_D(10.0f, 10.0f, 10.0f);
00024    static const Real     YAW_K_P = 0.5f;
00025    static const Real     YAW_K_D = 0.1f;
00026    static const CVector3 VEL_K_P(20.0f, 20.0f, 20.0f);
00027    static const CVector3 VEL_K_D(10.0f, 10.0f, 10.0f);
00028    static const Real     ROT_K_P = 0.5f;
00029    static const Real     ROT_K_D = 0.1f;
00030 
00031    /****************************************/
00032    /****************************************/
00033 
00034    CPointMass3DSpiriModel::CPointMass3DSpiriModel(CPointMass3DEngine& c_engine,
00035                                                   CSpiriEntity& c_spiri) :
00036       CPointMass3DQuadRotorModel(c_engine,
00037                                  c_spiri.GetEmbodiedEntity(),
00038                                  c_spiri.GetQuadRotorEntity(),
00039                                  BODY_HEIGHT,
00040                                  BODY_RADIUS,
00041                                  BODY_MASS,
00042                                  BODY_INERTIA,
00043                                  POS_K_P,
00044                                  POS_K_D,
00045                                  YAW_K_P,
00046                                  YAW_K_D,
00047                                  VEL_K_P,
00048                                  VEL_K_D,
00049                                  ROT_K_P,
00050                                  ROT_K_D) {
00051       /* Register anchor update methods */
00052       RegisterAnchorMethod(GetEmbodiedEntity().GetAnchor("rab"),
00053                            &CPointMass3DSpiriModel::UpdateRABAnchor);
00054       RegisterAnchorMethod(GetEmbodiedEntity().GetAnchor("camera"),
00055                            &CPointMass3DSpiriModel::UpdateCameraAnchor);
00056    }
00057 
00058    /****************************************/
00059    /****************************************/
00060 
00061    void CPointMass3DSpiriModel::UpdateRABAnchor(SAnchor& s_anchor) {
00062       s_anchor.Orientation = GetEmbodiedEntity().GetOriginAnchor().Orientation;
00063       s_anchor.Position = s_anchor.OffsetPosition;
00064       s_anchor.Position += GetEmbodiedEntity().GetOriginAnchor().Position;
00065    }
00066 
00067    /****************************************/
00068    /****************************************/
00069 
00070    void CPointMass3DSpiriModel::UpdateCameraAnchor(SAnchor& s_anchor) {
00071       s_anchor.Orientation = GetEmbodiedEntity().GetOriginAnchor().Orientation;
00072       s_anchor.Orientation *= s_anchor.OffsetOrientation;
00073       s_anchor.Position = s_anchor.OffsetPosition;
00074       s_anchor.Position.Rotate(s_anchor.Orientation);
00075       s_anchor.Position += GetEmbodiedEntity().GetOriginAnchor().Position;
00076    }
00077 
00078    /****************************************/
00079    /****************************************/
00080 
00081    REGISTER_STANDARD_POINTMASS3D_OPERATIONS_ON_ENTITY(CSpiriEntity, CPointMass3DSpiriModel);
00082 
00083    /****************************************/
00084    /****************************************/
00085 
00086 }
```

---

Generated on 10 Jul 2018 for ARGoS by 
 1.6.1
